# Supplementary material for: Snake phobia among the general population of Tamil Nadu, India
Source: PLoS Negl Trop Dis. 2024 Nov 5;18(11):e0012284. doi: 10.1371/journal.pntd.0012284 (PMC11567567; doi:10.1371/journal.pntd.0012284)
Supplement: S1 Questionnaire — (PDF) [file pntd.0012284.s001.pdf]

# Investigation of snake phobia in India

## Welcome

Dear Participant,

You have been invited to take part in this survey as you may or may not have a fear of snakes.

Fear of snakes is very common and many people across the world feel anxious about snakes. A phobia is an anxiety disorder that is characterised by a persistent and excessive fear in the presence of the object of the phobia. Animal phobia has been found to be one of the most prevalent forms of specific phobia.

We are researchers from the University of Reading, UK working with multiple institutions in India and we would like to determine the scale of snake phobia in India, especially as it is a country where snakebite is most prevalent.

Your participation in this survey will help us to better understand the impact of snakebites on mental health and wellbeing in snakebite endemic areas.

Many thanks for your time and support.

## Privacy Policy

This survey is in accordance with the Declaration of Helsinki and has been reviewed and approved by the University of Reading, School of Pharmacy Research Ethics Committee. The University of Reading, may use the information collected in this survey in several ways and data used for research purposes will be used in accordance with the General Data Protection Regulation (GDPR) 2016 and Data Protection Act (DPA) 2018. The results of this study may be used for conducting further research, statistical analysis and for scholarly purposes and may be shared with our academic collaborators. Any information that may be shared will be aggregated and anonymised to protect your identity. Information provided will be stored securely and deleted when no longer needed. Your participation in this research study is voluntary, you may choose not to participate. If you decide to participate you may withdraw at any time. By selecting “agree” below this indicates that you; understand the above information, voluntarily agree to participate, are a resident of India, are over the age of 18. If you do not wish to participate in the research study, please decline participation by selecting “disagree”. If you press ‘disagree’ you will not be able to participate. ✱ *Required*

- ○ Agree
- ○ Disagree

## Demographics

**First Name** ✱ *Required*

**Father's/spouse's/guardian's name** ✱ *Required*

**Gender** ✱ *Required*

- Male
- Female

**Age group** ✱ *Required*

- 18 - 25
- 26 - 30
- 31 - 35
- 36 - 40
- 41 - 45
- 46 - 50
- 51 - 55
- 56 - 60
- 61 - 65
- 66 - 70
- > 70

**Which district of Tamil Nadu are you from?** Please select 'Other Indian State - NOT FROM TAMIL NADU' if you do not live in Tamil Nadu. ✱ *Required*

- Ariyalur
- Chengalpattu
- Chennai
- Coimbatore
- Cuddalore
- Dharmapuri
- Dindigul
- Erode

- Kallakurichi
- Kanchipuram
- Kanyakumari
- Karur
- Krishnagiri
- Madurai
- Mayiladuthurai
- Nagapattinam
- Namakkal
- Nilgiris
- Perambalur
- Pudukkottai
- Ramanathapuram
- Ranipet
- Salem
- Sivaganga
- Tenkasi
- Thanjavur
- Theni
- Thoothukudi (Tuticorin)
- Tiruchirappalli
- Tirunelveli
- Tirupathur
- Tiruppur
- Tiruvallur
- Tiruvannamalai
- Tiruvarur
- Vellore
- Viluppuram
- Virudhunagar
- Other Indian State - NOT FROM TAMIL NADU

**What is your level of education?**
*✱ Required*

- No formal education
- Technical or vocational training
- Primary school (1st to 5th standard)
- Secondary school (6th to 12th standard)
- Tertiary/university level education (Bachelors, Masters, PhD and others)

# SNAQ-12

Please work through the next 12 statements. For each statement, indicate whether you agree or disagree.

If you agree with the statement answer with YES, if you disagree with the statement answer with NO.

Do not think too much about it – your initial responses are usually the best. Please answer all questions.

I would feel some anxiety holding a toy snake in my hand

நான் ஒரு பாம்பு பொம்மையை கையில் பிடிக்கவே பதட்டமடைவேன்

✱ *Required*

- ○ Yes
- ○ No

If a picture of a snake appears on the screen during a TV show/movie, I turn my head away

ஒரு பாம்பின் படம் சினிமாவிலோ அல்லது தொலைக்காட்சியிலோ வந்தாலே நான் அதை பார்க்காமல் என் தலையை திருப்பிக்கொள்வேன்

✱ *Required*

- ○ Yes
- ○ No

I dislike looking at pictures of snakes in magazines/newspapers

பாம்பின் படங்களை செய்தித்தாள்களிலோ அல்லது வேறு பத்திரிகைகளிலோ பார்ப்பதை நான் வெறுப்பேன்

✱ *Required*

- ○ Yes
- ○ No

I am terrified by the thought of touching a harmless snake

ஒரு விஷமில்லாத பாம்பை தொடவேண்டும் என நினைத்தாலே நான் பயப்படுவேன்

✱ *Required*

- ○ Yes
- ○ No

If someone says that there are snakes anywhere, I become alert

யாராவது எங்கேயாவது பாம்புகள் உள்ளது என்று சொன்னாலே நான் பதட்டமடைந்து உஷாராகி விடுவேன்

✱ Required

- Yes
- No

When I see a snake, I feel tense and restless

ஒரு பாம்பை நேரில் பார்த்தால் நான் மிகவும் பதட்டமடைவேன்

✱ Required

- Yes
- No

I feel unwell/sick when I see a snake

ஒரு பாம்பை நேரில் பார்த்தால் எனக்கு உடல்நிலை சரியில்லாமல் ஆகிவிடும்

✱ Required

- Yes
- No

The way snakes move is repulsive

பாம்புகள் நெளிந்து செல்வதைப் பார்த்தாலே எனக்கு ஒரு வெறுப்புணர்வு ஏற்படும்

✱ Required

- Yes
- No

If I came upon a snake in a forest/woods, I would probably run

ஒரு பாம்பை காட்டுக்குள் பார்த்தாலே நான் பயந்து ஓடுவேன்

✱ Required

- Yes
- No

I'm more afraid of snakes than any other animal

நான் மற்ற விலங்குகளைப் பார்த்து பயப்படுவதை விட பாம்புகளைப் பார்த்து அதிகம் பயப்படுவேன்

✱ *Required*

- ◌ Yes
- ◌ No

I would prefer not to finish a story if something about snakes was introduced

**ஒரு கதைப் படிக்கும்போது பாம்புகளைப் பற்றி ஏதேனும் வந்தால், நான் அந்த கதையைத் தொடர்ந்து படித்து முடிப்பதைத் தவிர்ப்பேன்**

✱ *Required*

- ◌ Yes
- ◌ No

Even if I was late for a very important appointment, the thought of snakes would stop me from taking a shortcut through an open field

**நான் ஒரு மிகவும் அவசரமான வேலைக்குச் சென்றாலும், பாம்புகள் இருக்குமோ என்ற அச்சத்தினால் குறுக்குப் பாதையை எடுப்பதைத் தவிர்ப்பேன்**

✱ *Required*

- ◌ Yes
- ◌ No

You have come to the end of the survey, thank you for your time.

Please select '**finish**' below to complete your participation.

If you have any questions about this study, please contact:

Prof. Sakthivel Vaiyapuri (s.vaiyapuri@reading.ac.uk)
Professor in Cardiovascular & Venom Pharmacology, School of Pharmacy, University of Reading, UK

Or

Anika Salim (anika.salim@pgr.reading.ac.uk)
PhD researcher, School of Pharmacy, University of Reading, UK

## Farewell

**You have come to the end of the survey, thank you for your time.**

You must consent to participate in this survey.

If you have mistakenly pressed 'disagree' please reload the link and press 'agree' to participate.
